# Supplementary material for: A Humanized Yeast Phenomic Model of Deoxycytidine Kinase to Predict Genetic Buffering of Nucleoside Analog Cytotoxicity
Source: Genes (Basel). 2019 Sep 30;10(10):770. doi: 10.3390/genes10100770 (PMC6826991; doi:10.3390/genes10100770)
Supplement: Supplementary file 1 [file genes-10-00770-s001.zip › Additional_Files/Additional_File7_GOTermHeatmaps.zip!/Additional_File7_GOTermHeatmaps/mitochondrial_ribosome.pdf]

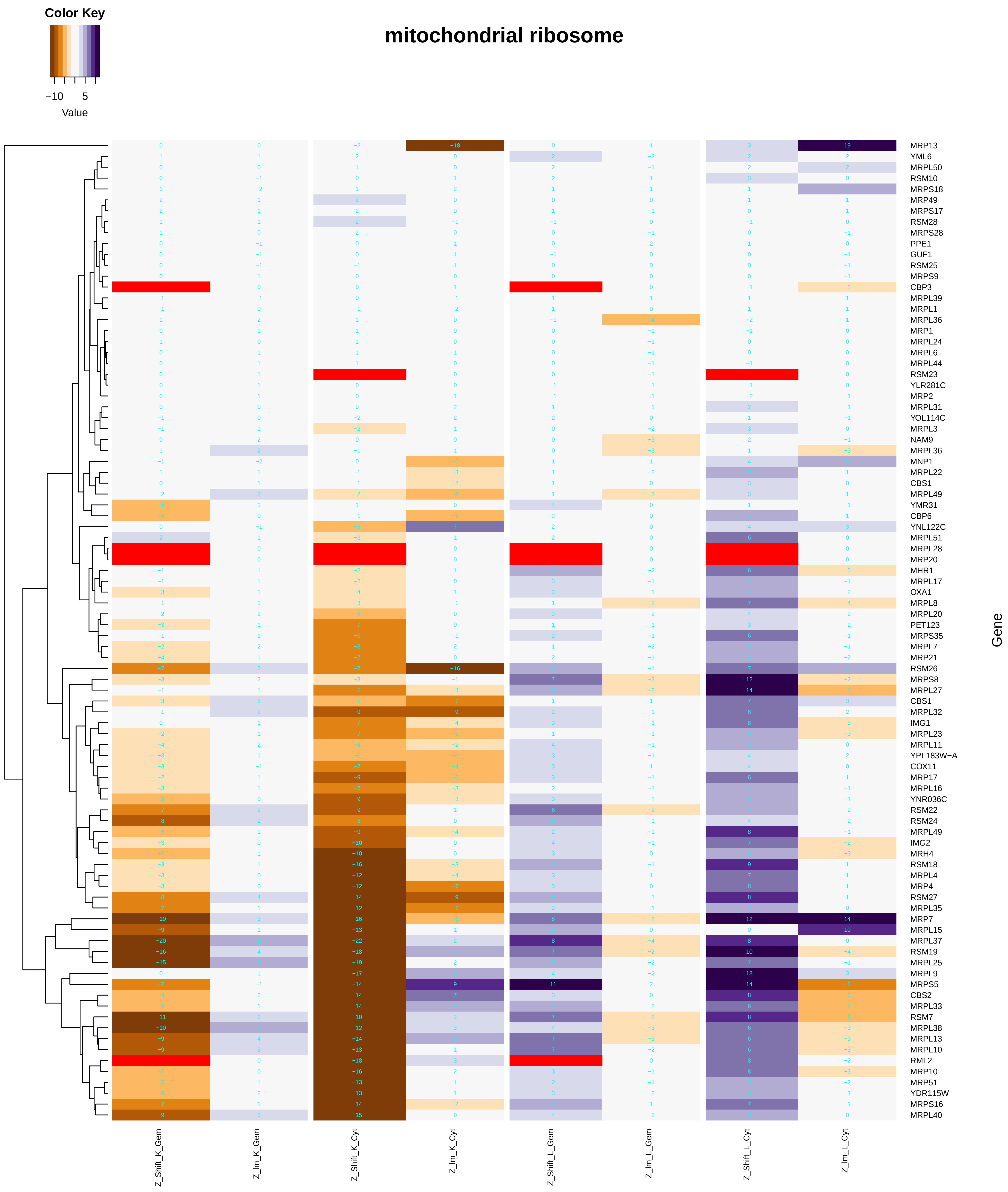
mitochondrial ribosome
Color Key
−10
5
Value
Gene
MRP13
YML6
MRPL50
RSM10
MRPS18
MRP49
MRPS17
RSM28
MRPS28
PPE1
GUF1
RSM25
MRPS9
CBP3
MRPL39
MRPL1
MRPL36
MRP1
MRPL24
MRPL6
MRPL44
RSM23
YLR281C
MRP2
MRPL31
YOL114C
MRPL3
NAM9
MRPL36
MNP1
MRPL22
CBS1
MRPL49
YMR31
CBP6
YNL122C
MRPL51
MRPL28
MRP20
MHR1
MRPL17
OXA1
MRPL8
MRPL20
PET123
MRPS35
MRPL7
MRPL21
RSM26
MRPS8
MRPL27
CBS1
MRPL32
IMG1
MRPL23
MRPL11
YPL183W−A
COX11
MRP17
MRPL16
YNR036C
RSM22
RSM24
MRPL49
IMG2
MRH4
RSM18
MRPL4
MRP4
RSM27
MRPL35
MRP7
MRPL15
MRPL37
RSM19
MRPL25
MRPL9
MRPS5
CBS2
MRPL33
RSM7
MRPL38
MRPL13
MRPL10
RML2
MRP10
MRP51
YDR115W
MRPS16
MRPL40
Z_Shift_K_Gem
Z_Im_K_Gem
Z_Shift_K_Cyt
Z_Im_K_Cyt
Z_Shift_L_Gem
Z_Im_L_Gem
Z_Shift_L_Cyt
Z_Im_L_Cyt
